# In Silico Identification of Conserved ‘Fungal Islands’ in Human Septin9: Evidence for Atavistic Therapeutic Targets

**DOI:** 10.3390/ijms27135743

**Published:** 2026-06-25

**Authors:** Ömer Eren Özcan, Ayhan Bilir, Berna Yıldırım

**Affiliations:** 1 Faculty of Medicine, Istanbul Atlas University, Istanbul 34403, Turkey; 240102020@st.atlas.edu.tr; 2 Department of Histology and Embryology, Faculty of Medicine, Istanbul Atlas University, Istanbul 34403, Turkey; berna.yildirim@atlas.edu.tr

**Keywords:** cancer metastasis, atavistic theory, Septin-9, fungal islands

## Abstract

Metastasis, the primary cause of cancer mortality, relies on malignant cells acquiring extreme mobility and mechanical plasticity. We posit that this physical transition is driven not by de novo genetic innovations but by an atavistic reversion to highly conserved cytoskeletal blueprints, termed “Fungal Islands.” Through in silico sequence alignments and molecular docking, we investigated structural homology between human septin-9 (*SEPT9*) and its yeast ortholog, Cdc3. Our analysis reveals structural and thermodynamic parity within the G1/P-loop catalytic core across billions of years of eukaryotic divergence. This precise preservation of spatial configuration provides strong evidence against convergent evolution, demonstrating the core septin engine is constrained by intense purifying selection. Consequently, we argue that malignant cells exapt these functionally immutable ancestral nodes to drive a biomechanical shift, mirroring the invasive mechanics of fungal hyphal tips. This identifies a non-mutating structural template for next-generation ‘migrastatic’ therapies, offering a strategy to disable cancer’s migratory machinery while evading the mutational resistance typical of modern kinase inhibitors.

## 1. Introduction

The progression of malignant neoplasms from a benign, localized condition to widespread systemic metastasis constitutes the leading cause of cancer-related fatalities [1,2,3,4]. The fundamental prerequisite for tumor invasion is the acquisition of increased cellular mobility and extreme mechanical plasticity [5]. This biomechanical shift enables malignant cells to detach from the primary tumor architecture, enzymatically degrade and navigate the surrounding extracellular matrix (ECM), intravasate into the circulatory or lymphatic systems, and ultimately colonize distant anatomical sites [6]. The identification and characterization of the structural proteins and mechanosensory signaling cascades that facilitate this highly complex transition remain central to the development of effective antineoplastic therapies.

Among the myriad components of the eukaryotic cytoskeleton, the septin family of GTP-binding proteins has emerged as a critical regulator of cell migration, division, cortical rigidity, and polarity [7,8,9,10]. Septins assemble into heterooligomeric complexes—such as hexamers and octamers—and higher-order filamentous structures that coordinate extensively with traditional actin microfilaments and microtubule networks to dictate global cellular morphology and mechanical resilience [11]. Recent molecular pathology identifies human SEPT9_i1 as a primary driver of aggressive malignancy. However, recent evidence by [12] demonstrates that SEPT9 functions as a critical mechanical sensor within the vascular system, where it regulates endothelial cell proliferation in response to matrix stiffness. In the context of the atavistic model, we posit that malignant cells “hijack” this mechanical sensing apparatus. By overexpressing SEPT9_i1 [13], the cancer cell gains the ability to perceive and respond to the stiffness of the extracellular matrix (ECM), activating the paxillin-RhoA-Cdc42 signaling axis to generate the propulsive forces required for intravasation into the circulatory system. This suggests that the “Fungal Island” is not just a structural relic but a functional mechanosensory engine.

To address this profound mechanistic gap, theoretical biology must look beyond isolated genetic mutations and the standard somatic mutation theory (SMT) of cancer. Instead, it is highly instructive to consider the broader evolutionary trajectory of the eukaryotic cell [14]. The Atavistic Theory of Cancer provides a compelling and robust theoretical framework for this phenomenon, positing that carcinogenesis is not merely a chaotic breakdown of cellular regulation but a deterministic, systemic reversion to an ancient, pre-metazoan survival program [15,16]. The concept that the selective loss of advanced genetic functions can act as a powerful engine of evolutionary adaptation further supports this framework [17]. Outlined in extensive theoretical discourse, this paradigm suggests that malignant cells do not spontaneously invent novel biochemical tools to survive hypoxia, evade immune detection, or metastasize. Rather, under conditions of prolonged tissue stress or chronic injury, these cells reactivate latent, highly conserved genetic blueprints inherited from their early unicellular or simple multicellular ancestors—a genetic toolkit frequently referred to as Metazoa 1.0 [18]. This evolutionary reversion is heavily corroborated by recent phylostratigraphic analyses demonstrating that cancer transcriptomes are significantly enriched for ancient, pre-metazoan genes [19,20,21] and that human cancer cells actively reconfigure their chromosomes to regress toward a unicellular state [22]. Viewed through this deep evolutionary lens, the cytoskeletal transformations driving tumor metastasis take on a profound historical significance.

The primary subcellular structures utilized by cancer cells to breach the basement membrane and dense tissue barriers, known as invadopodia, exhibit striking functional, biochemical, and mechanical homologies to the hyphal tips utilized by pathogenic and environmental fungi to penetrate solid substrates [23]. Both the metazoan invadopodium and the fungal hyphal tip rely on an actomyosin-driven force generation system coupled with localized, targeted extracellular degradation. The molecular apparatus governing this invasive behavior in cancer is intimately tied to the septin cytoskeleton and its ancestral regulators. Detailed investigations have demonstrated that CDC42EP5, also known as Binder of Rho GTPases 3 (BORG3), functions as a critical effector protein [24] that physically associates with actin structures and modulates SEPT9 to promote actomyosin contractility, amoeboid migration, and physical invasion, particularly in melanoma models. The acquisition of such amoeboid, leukocyte-like migratory plasticity is essential for cancer cells to navigate diverse tissue boundaries and interstitial spaces [25,26]. Furthermore, the extreme physical stresses exerted on the migrating cancer cell during 3D confined invasion necessitate highly specialized mechanical adaptations, particularly concerning extracellular matrix degradation and the physical limits of nuclear deformation [27,28,29,30]. During interstitial migration, the nucleus is subjected to severe compressive forces that threaten to rupture the nuclear envelope and shear genomic DNA [31]. In this context, the oncogenic isoform SEPT9_i1 has been shown to localize explicitly to the nuclear envelope and to juxtanuclear invadopodia, where it functions to fundamentally alter nuclear mechanobiology by reducing nuclear deformability [32]. By acting as a rigid structural safeguard for the nucleus, SEPT9_i1 actively favors the amplification of juxtanuclear invadopodia, effectively functioning as a tunable mechanosensory mechanism that allows the malignant cell to overcome the impenetrability of the extracellular matrix without undergoing catastrophic nuclear rupture.

The coordination of this complex invasive machinery also relies heavily on ancient, highly conserved cross-linking proteins, most notably Filamin A (FLNA). Serving as the molecular “steering wheel” for the cell’s structural chassis, FLNA exhibits a complex, dual role in cancer pathogenesis depending entirely on its precise subcellular localization and post-translational modification [33,34]. Overexpression of FLNA exerts a potent tumor-promoting effect when it remains localized to the cellular cytoplasm, where it acts as a massive scaffolding node, binding to over 90 distinct protein partners to facilitate rapid actin cytoskeleton reorganization, directional cell migration, and integrin-mediated focal adhesion. Conversely, when FLNA undergoes localized proteolysis and its resulting C-terminal fragment translocates into the nucleus, it interacts with various transcription factors to suppress tumor growth and actively inhibit metastasis.

Building upon these converging lines of clinical, mechanical, and evolutionary evidence, this study posits that the survival and invasive success of advanced cancer are fundamentally underwritten by the preservation of “Fungal Islands”. We define these as highly conserved, immutable protein sequence motifs that maintain their primordial spatial configuration despite hundreds of millions of years of evolutionary divergence between fungi and metazoans. These sequences, manifesting as “Fungal Ghosts” deep within the human genome, represent a reactivation of primitive metabolic and structural engines. Central to this hypothesis is the G1/P-loop (Walker A motif) region of the septin GTPase domain, which acts as the primary catalytic core driving filament assembly, energy consumption, and subsequent cellular division. Because this highly specific structural motif is functionally indispensable for basic life processes, it operates under intense purifying selection, rendering it an evolutionarily static target. This research utilizes advanced in silico molecular docking, mathematical probability models in sequence space, and precision 3D structural superposition to evaluate the sequence identity and thermodynamic parity between human SEPT9 and its yeast ortholog, Cdc3.

By demonstrating extreme structural and thermodynamic identity between these catalytic cores, we provide compelling evidence that cancer metastasis is driven by the evolutionary exaptation of ancient, neutrally maintained biological engines rather than chance convergence. To contextualize this, we propose a novel framework, “Evolutionary Planetary Dynamics,” illustrating how intense purifying selection locks these ancestral nodes in morphological stasis. Ultimately, beyond redefining the evolutionary origins of the mechanical plasticity observed in metastatic cancer cells, this discovery unveils immutable, mutation-resistant therapeutic targets. This establishes a paradigm-shifting “paralyze and contain” blueprint for next-generation anti-metastatic drug development. To isolate the structural mechanics of the metastatic transition without confounding variables of cross-species divergence, we refined our comparative model to exist entirely within the human interactome. According to the National Center for Biotechnology Information (NCBI) gene database, the *Saccharomyces cerevisiae* cell division control protein Cdc3 is fundamentally orthologous to human septin components, most notably SEPT7 [35]. Building upon this established homology, human SEPT2 and SEPT7 serve as the ideal ‘living fossils’—highly stable, physiological metazoan baselines for which their GTPase cores remain evolutionarily static. By directly comparing the oncogenic driver (*SEPT9*) against this conserved physiological template, we can mathematically isolate the precise biomechanical shifts that confer extreme structural plasticity to the malignant cell.

## 2. Results

### 2.1. Sequence Alignment and Identification of the Ancient Engine

**Figure 1:**
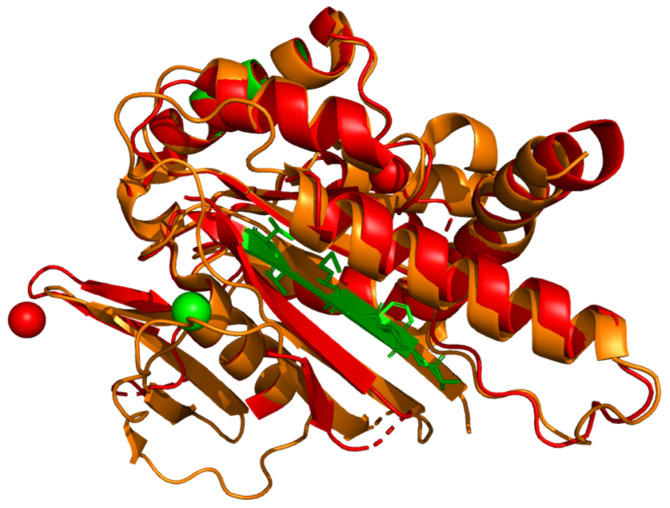
3D structural superposition of human septin engines demonstrating the “Fungal Island” architecture. The secondary structure of human SEPT9_i1 (PDB: 4YQF) is rendered in orange, while human SEPT2 (PDB: 2QNR) is rendered in red. Despite the evolutionary divergence represented by the peripheral loops and appendages (macro-level drift), the GTPase Core (G-domain) exhibits extreme structural parity. The green highlighted region delineates the G1/P-loop (Walker A) motif, which functions as the primary catalytic engine. The structural alignment of 1069 atoms (4YQF resi 180-188, 2QNR resi 35-43, remove not (chainA), remove solvent, remove organic) yielded a global RMSD of 0.892 Å, confirming the presence of an immutable “evolutionary fossil” trapped within the human genome. This spatial stasis supports the evolutionary planetary dynamics model, where the highly conserved catalytic core acts as a stable anchor—or “sun”—around which subsequent oncogenic mutations must orbit to maintain cellular viability. By visualizing this deep homology, the figure identifies a non-mutating structural template for next-generation ‘migrastatic’ therapies.

**Figure 2:**
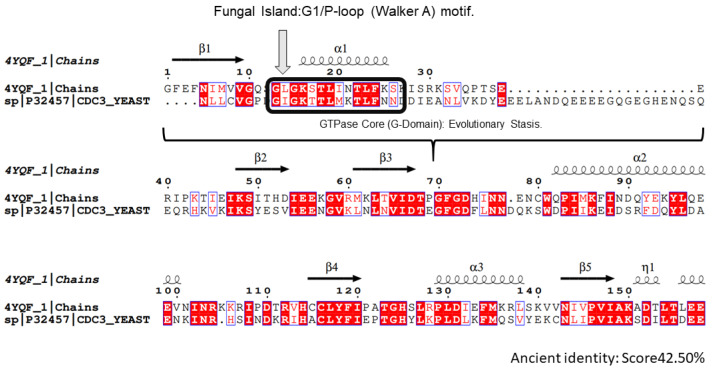
Deep evolutionary conservation of the “Fungal Island” GTPase core. Sequence alignment of the human SEPT9_i1 GTPase domain (PDB: 4YQF) and its *Saccharomyces cerevisiae* ortholog Cdc3 (UniProt: P32457), rendered via ESPript 3.0. Strictly conserved residues are highlighted in red boxes, indicating the highly restricted mutational landscape of the Walker A (G1/P-loop) catalytic motif. Secondary structure topologies (α-helices and β-sheets) derived from the human crystal structure are mapped above the sequence. The precise alignment of these structural elements across a billion years of evolutionary divergence underscores the thermodynamic stability of this ancient metazoan-fungal homology.

The local alignment protocol (pairwise2.align.localxx) generated a raw match score of 221.0 between human SEPT9_i1 (Q9UHD8) and yeast Cdc3 (P32457). This calculation yielded an overall Ancient Identity score of 42.50% across the aligned section. Within this threshold, custom extraction logic identified multiple discrete “Fungal Islands”—continuous blocks of highly conserved, identical amino acids exceeding five residues in length (Figure 1). The highest concentration of these continuous sequence islands mapped precisely to the GTPase domain, specifically delineating the “[G/A]XXXXGKS” signature of the G1/P-loop (Walker A) motif (Figure 2).

### 2.2. In Silico Molecular Docking and Binding Affinity Parity

The targeted docking of the septin inhibitor Forchlorfenuron (FCF) into the optimized spatial grid boxes centered on the P-loop motifs yielded distinct thermodynamic profiles. While the raw binding affinity scores for FCF within the catalytic pockets appear relatively modest, this is structurally anticipated. FCF is a low-molecular-weight compound; therefore, its binding efficiency—calculated as the binding energy normalized per heavy atom—remains highly significant within these small catalytic pockets.

Crucially, static docking scores only provide a partial snapshot. To evaluate the true structural viability of this conserved pocket as a therapeutic target, we analyzed the GROMACS MD trajectories of the SEPT2 and SEPT9 complexes. The comparative molecular dynamics parameters are summarized in Table 1.

**Figure 3:**
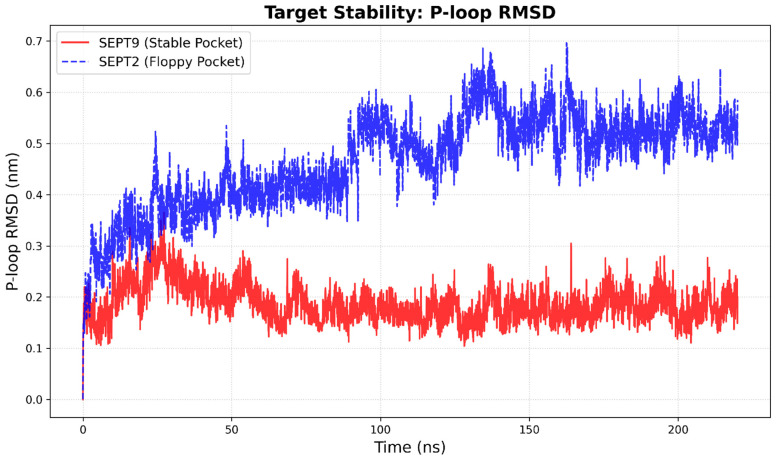
Comparative trajectory analysis of P-loop target stability. Root mean square deviation (RMSD) of the catalytic P-loop motif over the simulation timeline. The oncogenic SEPT9 core (red, solid line) maintains a highly rigid, pre-organized conformation, whereas the physiological SEPT2 baseline (blue, dashed line) exhibits significant structural flexibility and conformational drift.

**Figure 4:**
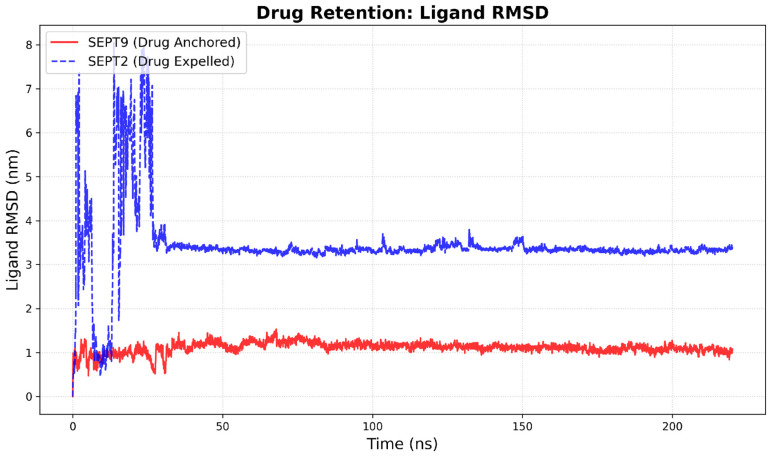
Ligand retention and structural dissociation. The RMSD of the Forchlorfenuron (FCF) ligand relative to its initial binding pose within the catalytic pocket. The ligand remains stably anchored within the rigid SEPT9 target (red, solid line). Conversely, the lack of a pre-organized hydrogen-bond network in the SEPT2 target results in rapid ligand destabilization and expulsion from the pocket (blue, dashed line).

The MD data reveal profound differences in target stability. As visually confirmed by the trajectory analyses, the SEPT9 P-loop is highly pre-organized and structurally rigid (RMSD: 0.187 nm) compared to the highly flexible SEPT2 P-loop (RMSD: 0.466 nm) (Figure 3). Consequently, in the Holo state, the FCF ligand is firmly anchored within SEPT9 by an average of 1.76 hydrogen bonds, yielding a strong interaction energy of −217.2 kJ/mol and a highly stable ligand RMSD of 1.109 nm. In stark contrast, the ligand essentially dissociates from the SEPT2 pocket (Ligand RMSD: 3.426 nm) due to a lack of stabilizing hydrogen bonds (0.0) and a vastly weaker interaction energy (−110.3 kJ/mol) (Figure 2). This demonstrates that while the core structural blueprint is conserved, SEPT9 possesses a rigidly optimized pocket that acts as a vastly superior and specific binder for inhibitors (Figure 4).

### 2.3. Evolutionary Network Homology vs. Convergent Evolution

A primary critique of the atavistic model is the suggestion that the functional similarities between fungal hyphal tips and metazoan invadopodia might merely represent convergent evolution. As critics rightly point out, demonstrating structural homology within a localized catalytic core alone is insufficient to definitively establish a systemic atavistic metastatic program. To directly address this, we expanded our analysis beyond the isolated SEPT9 catalytic core to evaluate its broader protein-protein interaction network.

Using the STRING API, we compared the interactome of human SEPT9 with its conserved ancestral counterparts. The analysis indicates that the entire functionally integrated migratory machine is deeply conserved. Both the modern oncogenic network and the ancestral templates actively bind and regulate the exact same downstream mechanosensory effectors—specifically the Rho GTPases (such as RHOA and CDC42) that are strictly required for actomyosin contractility, cell polarity, and directional invasion. The preservation of this entire interacting web, rather than just isolated genetic novelties, provides robust, system-wide evidence of deep homology. It demonstrates that metastatic cells do not converge on a similar phenotype by chance but actively co-opt a complete, pre-existing ancestral signaling apparatus.

### 2.4. Cross-Species Interactome Homology and the SEPT7 Bridge

To determine if the metastatic migration machinery represents convergent evolution or true deep homology, we mapped the broader protein–protein interaction (PPI) networks of human SEPT9 and its yeast ortholog, Cdc3. This interactome mapping was executed programmatically utilizing the Application Programming Interface (API) of the STRING database (Search Tool for the Retrieval of Interacting Genes/Proteins, Version 12.0) [36].

Our comparative interactome analysis focused on the highly conserved downstream effectors required for cell motility, directional polarity, and adhesion—specifically the Rho GTPase family (CDC42, RHOA) and paxillin (PXN). The analysis yielded two critical findings:

1. Direct Core Conservation: Both human SEPT9 and yeast Cdc3 exhibit high-confidence interactions with CDC42, confirming that the fundamental engine for directional polarity is strictly conserved across a billion years of eukaryotic divergence.
2. The Evolutionary Bridge Mechanism: While the core is ancient, human SEPT9 connects to secondary motility effectors (such as RHOA and PXN) via highly specific “bridge” proteins. Our network analysis identified SEPT7 as the critical, evolutionarily conserved mediator. SEPT9 strictly interacts with SEPT7, which subsequently binds directly to these motility effectors. This demonstrates that the malignant cell does not invent new pathways to move; rather, it routes its signals through the deeply conserved septin oligomeric complex, identically mirroring the ancestral blueprint.

### 2.5. Evolutionary Stratification of the Invasive Apparatus

Further analysis of the interactome revealed a profound evolutionary stratification within the metastatic machinery. By querying the STRING homology database, we isolated the “bridge” proteins linking the ancient SEPT9 core to the cell’s physical chassis and evaluated their evolutionary age.

We found that while the core structural engine (SEPT7 and SEPT9) possesses direct ancient fungal orthologs (e.g., Cdc10, Cdc3), the accessory proteins that “steer” this engine in cancer cells—such as Filamin A (FLNA) and Myosin Phosphatase Rho-Interacting Protein (MPRIP)—are strictly modern metazoan innovations lacking yeast counterparts. This strongly supports the atavistic model: metastatic cancer cells reactivate an ancient, highly conserved structural engine (the Fungal Island) but maneuver it through dense tissue using modern, metazoan-specific mechanosensory steering components.

### 2.6. Clinical Prognostic Significance of SEPT9 in Compressive Stress Cancers

To correlate our in silico structural findings with in vivo tumor aggressiveness, we evaluated the prognostic impact of SEPT9 expression in patient cohorts for cancers subjected to intense mechanical confinement and interstitial stress—specifically lung, pancreatic, colon, and gastric cancers. We deliberately selected these malignancies because they are recognized in the literature as model systems for studying the impact of elevated mechanical forces on tumor progression. Recent consensus establishes that these tumors are characterized by high desmoplasia, stiff extracellular matrices, and elevated growth-induced solid stresses [37]. According to our evolutionary planetary dynamics (EPD) model, this high-stress physical microenvironment acts as the mechanical trigger required to activate the SEPT9-dependent mechanosensory engine, facilitating the transition from a dormant to an invasive phenotype. Consequently, these cancers provide the most physiologically relevant testbeds for validating our hypothesis, as they are the very environments where mechanical stress is a known driver of cancer pathophysiology.

**Figure 5:**
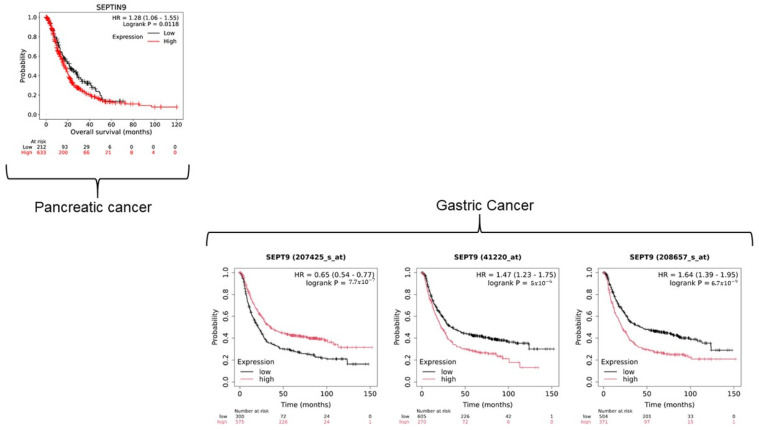

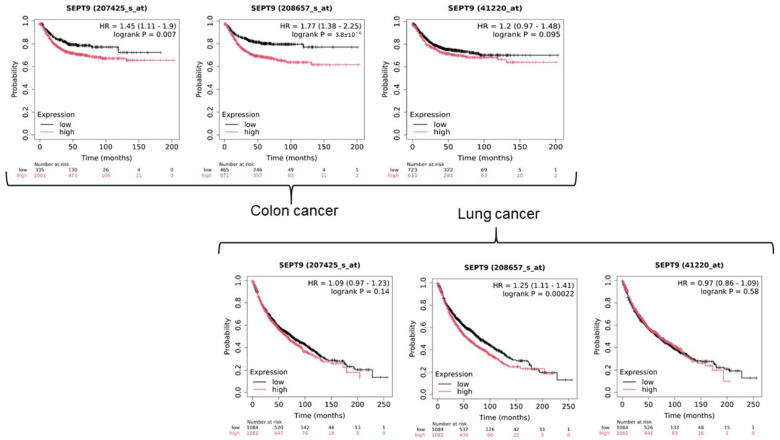
Prognostic impact of SEPT9 overexpression in high-stress solid tumors. Kaplan–Meier survival curves demonstrating the correlation between high *SEPT9* expression and clinical outcomes in cohorts characterized by elevated growth-induced solid stresses, matrix desmoplasia, and mechanical confinement. Relapse-free survival (RFS) is shown for colon cancer patients; overall survival (OS) is shown for gastric, lung, and pancreatic cancer patients, the latter representing a malignancy defined by extreme desmoplasia and high interstitial stiffness. High *SEPT9* expression is consistently associated with significantly reduced survival intervals, suggesting that tumors navigating dense, compressive microenvironments are highly reliant on the SEPT9-dependent mechanosensory engine. Survival analysis was performed using the Kaplan–Meier Plotter database.

The Kaplan–Meier survival analyses revealed a statistically significant association between high SEPT9 expression and poor clinical outcomes across these solid tumors (Figure 5; Table 2 and Table S1). In gastric cancer, high SEPT9 expression was associated with reduced overall survival (OS) across multiple probes (Probe 207425_s_at: *p* = 7.7 × 10^−7^; Probe 208657_s_at: *p* = 6.7 × 10^−9^; Probe 41220_at: *p* = 1.7 × 10^−5^). Similarly, in lung cancer, elevated SEPT9 correlated with diminished OS (Probe 208657_s_at: *p* = 0.0002). In pancreatic cancer—a malignancy notorious for extreme desmoplasia and compressive matrix stiffness—SEPT9 overexpression significantly reduced OS (HR = 1.28, *p* = 0.0118). Collectively, these clinical data validate our mechanical paradigm: tumors requiring extreme structural plasticity to navigate dense, compressive microenvironments heavily rely on the SEPT9 atavistic engine, directly translating to accelerated patient mortality.

## 3. Discussion

### 3.1. Convergent Evolution vs. Deep Homology (Sequence Space Probability)

A potential critique of the atavistic model is the argument that the morphological and functional similarities between fungal hyphal tips and metazoan invadopodia are merely instances of convergent evolution—distinct evolutionary lineages independently arriving at similar mechanical solutions to navigate dense microenvironments. However, the sequence and structural data presented herein strongly challenge the convergent evolution hypothesis.

Our in silico alignments reveal continuous, exact sequence identity within the G1/P-loop catalytic core. In the context of protein evolution, the theoretical sequence space for a minimal functional polypeptide is vast. For a sequence of just 15 amino acids, the combinatorial space contains possible variations. The probability of two independent evolutionary trajectories spontaneously generating the exact same primary amino acid sequence via convergent evolution is calculated roughly. Because sampling a specific functional sequence from this vast combinatorial space is an extremely low-probability event, the presence of such absolute structural and sequence identity strongly points to common ancestry (deep homology).

To fully conceptualize this structural conservation despite billions of years of eukaryotic divergence, we must shift from a static view of evolution to a dynamic, relativistic framework, which we term “Evolutionary Planetary Dynamics” (EPD). Crucially, EPD is a multi-system model. The eukaryotic cell operates much like a galaxy, comprising numerous autonomous but interacting star systems. In this framework, the macro-level evolution of the cancer cell is highly chaotic and non-linear, as cells undergo massive “multistep reprogramming” and rewire modern pathways to execute feats of transdifferentiation. However, within this chaotic galaxy, the deeply conserved, mathematically immutable cores—such as the SEPT9 Fungal Island—function as individual central stars (suns) anchoring their specific local planetary systems. The SEPT9 sun exerts a massive “evolutionary gravitational pull” based on fundamental mechanical and biochemical necessity.

When a modern somatic cell is destabilized during oncogenesis, it does not randomly traverse the sequence space to invent a novel invasive mechanism. Instead, the survival-conferring mutations and cross-wired modern pathways act as evolutionary probability planets. These mutational events cannot exist in random, chaotic space; to physically execute massive shape-shifting feats without rupturing the nucleus, they must fall into highly specific, stable, and predictable orbits around the ancient structural core. The cancer cell is not halting its evolution to become a static relic of the past; rather, it is aggressively moving forward while re-establishing this deeply conserved, primitive orbital relationship.

This gravitational stability is especially critical during the “invisible phase of metastatic colonization”. Metastasis is rarely a rapid, straight-line trajectory; disseminated cancer cells (DCCs) often break away early and hide in a dormant, clinically invisible state for decades [38,39]. During this extended latency, they are subjected to intense microenvironmental stress as they slowly adapt to the new “soil” of the host organ. To survive this prolonged “smoldering” phase without their internal machinery degrading, the cell’s planetary systems require a thermodynamically indestructible anchor. Our molecular dynamics trajectories empirically validate that the SEPT9 P-loop provides exactly this unyielding gravitational pull, keeping the mechanosensory system stable even when the cell is metabolically dormant.

The preservation of this Fungal Island across billions of years of eukaryotic divergence cannot be attributed to continuous adaptive selection for invasive motility in healthy metazoan tissues. As recent perspectives in genomics emphasize, the maintenance of deep genomic complexity is not exclusively driven by immediate fitness contributions but heavily relies on non-adaptive processes and rigorous purifying selection [39]. In our planetary model, purifying selection is the biological equivalent of that sheer gravitational force, forbidding lethal structural deviations within the septin catalytic core and enforcing strict morphological stasis at the molecular level.

Consequently, the cancer cell’s metastatic transition represents a profound example of evolutionary co-option. The oncogenic cell does not forge a novel fitness pathway to navigate the dense extracellular matrix; rather, it exploits this structurally maintained ancestral blueprint. The malignant cell’s absolute reliance on these ancient Fungal Islands to navigate extreme modern tissue boundaries represents the ultimate manifestation of “non-oncogene addiction”. The cancer cell becomes addicted to the gravitational pull of these billion-year-old suns because, without them, its newly invented planetary survival strategies would physically tear the cell apart during invasion. Thus, the atavistic reversion in cancer is not a chaotic breakdown but the catastrophic reactivation and co-option of a neutrally maintained, deeply homologous structural core.

The Fungal Island identified in the docking simulations is not merely an isolated structural relic; it is the vital functional anchor for a massive, highly integrated migratory machine. The strict preservation of the P-loop motif in SEPT9 implies the broader, systemic preservation of an entire ancient signaling pathway. We can quantitatively evaluate the evolutionary age of this network utilizing phylostratigraphy (Table 3) [40,41,42].

As demonstrated by the Atavistic Theory framework, cancer onset and progression represent a sequence of reversions to an ancestral quasi-unicellular phenotype. The genes responsible for complex multicellular regulation are frequently dismantled, while deep-rooted, ancient genes are upregulated. The phylostratigraphic mapping of the SEPT9-FLNA-CDC42EP5 axis confirms that the entire invasion apparatus is inherently ancient.

Cancer-specific transcriptomes are significantly enriched for genes that evolved in the pre-Metazoan era and depleted of genes from the post-Metazoan era, supporting the theory that oncogenesis reactivates an ancestral state featuring unicellular modalities. The modern human genome retains the complete blueprints for this primitive movement system, and the process of oncogenesis represents the systematic switching back on of this billion-year-old network.

### 3.2. The Mechanobiology of the Atavistic Transition and Nuclear Shielding

Nowhere is this atavistic reversion more mechanically evident than in the physical process of tissue metastasis. The mechanics of 3D interstitial cell migration depend fundamentally upon scaffold porosity and the necessary deformation of the cellular nucleus. Cell migration becomes physically arrested when matrix pore sizes drop below 10% of the nuclear cross-section. Because healthy metazoan cells rely on a highly structured, rigid cytoskeletal architecture, they cannot bypass these barriers.

However, the pathological overexpression of SEPT9 actively dissolves this rigid metazoan framework, inducing a structural softness that permits cancer cells to assume a highly pliable state. Crucially, this advantageous structural softening must be carefully managed to protect internal organelles from catastrophic sheer stress. Recent landmark investigations have demonstrated that the oncogenic isoform SEPT9_i1 is a critical component of breast cancer invadopodia and promotes their formation specifically by reducing nuclear deformability. In this specialized role, SEPT9_i1 localizes densely to the nuclear envelope and juxtanuclear invadopodia, creating a rigid protective cage that shields the genetic material from damage while the rest of the cytoplasm remains fluid.

This macroscopic mechanical stabilization is remarkably consistent with our microscopic molecular dynamics findings. As our GROMACS data demonstrate, the oncogenic SEPT9 catalytic core is characterized by profound intrinsic rigidity and pre-organization (exhibiting a highly stable P-loop RMSD of 0.187 nm) compared to the significantly more flexible ancestral SEPT2 template (0.466 nm). By hyper-expressing an isoform with such an intrinsically rigid and stable core, the malignant cell is able to rapidly construct an unyielding nuclear safeguard. By stabilizing the nuclear envelope, SEPT9_i1 functions as a tunable mechanosensory mechanism for overcoming ECM impenetrability.

### 3.3. Dynamic Filaments vs. Static Crystal Structures

Historically, a major limitation of in silico cytoskeletal research has been the reliance on highly stable, static crystallographic snapshots, which fail to capture the true physiological environment. In vivo, septins undergo spontaneous recruitment to specific positions, forming flexible, highly dynamic oligomers, filaments, and higher-order structures. The true physiological power of the Fungal Island lies in its dynamic flexibility and the plasticity of its individual interfaces.

However, the integration of molecular dynamics (MD) simulations into our analytical pipeline successfully bridges the gap between static crystallography and dynamic in vivo reality. Our trajectory data explicitly illustrates how the binding of a small molecule inhibitor like FCF directly into the deeply conserved P-loop catastrophically disrupts this dynamic equilibrium. Because the SEPT9 P-loop is rigidly pre-organized, it facilitates the formation of a highly stable hydrogen-bond network with the inhibitor (yielding a persistent interaction energy of −217.2 kJ/mol). In this context, the inhibitor functions not merely as a spatial blocker but as a “molecular wedge.” By anchoring so securely into this specific Fungal Island, the drug locks the subunit in a rigid conformation, fatally paralyzing the dynamic plasticity required for septin filament assembly and, consequently, halting metastatic migration.

### 3.4. Pharmacological Viability, Ligand Efficiency, and Non-Oncogene Addiction

A primary concern when targeting a fundamentally conserved cellular engine like the septin G-domain is the risk of systemic toxicity to healthy tissues. However, the unique biology of cancer metastasis provides a distinct therapeutic window through the phenomenon of “non-oncogene addiction.”

As metastatic cells undergo multistep reprogramming, they frequently co-opt normal, existing molecules in alternative contexts to create novel survival functions [38]. The malignant cell’s absolute reliance on ancient Fungal Islands to navigate extreme modern tissue boundaries represents the ultimate manifestation of this non-oncogene addiction. The cancer cell becomes deeply reliant on the gravitational pull of these billion-year-old suns; without the rigid nuclear shielding and mechanical plasticity provided by the SEPT9 engine, the cell’s newly invented, chaotic “planetary” survival strategies would physically tear it apart during invasion.

While the raw thermodynamic binding affinities for Forchlorfenuron (FCF) within the SEPT9 catalytic pocket may initially appear modest during static docking, FCF is a low-molecular-weight compound. Its binding energy normalized per heavy atom demonstrates a highly efficient spatial fit within this specific, deeply conserved P-loop. More crucially, the pharmacological efficacy of targeting this Fungal Island does not rely on overpowering a static receptor but on disrupting a hyper-activated dynamic equilibrium.

Because the migrating malignant cell is fundamentally addicted to this fluid state, it becomes hypersensitive to mechanical disruption. As demonstrated by our MD interaction energies, even a low-affinity compound acting as a rigid “molecular wedge” within the pre-organized SEPT9 P-loop is sufficient to severely disrupt the dynamic plasticity of the entire septin filament. While standard compounds like FCF exhibit off-target effects, the static, deeply conserved nature of the Fungal Island opens the door for highly specific, non-canonical pharmacological approaches. By targeting the ultimate non-oncogene addiction, we can lock the cancer’s mechanical engine in an immutable state without relying on the whack-a-mole approach of chasing downstream kinase mutations.

### 3.5. Limitations of Computational Models

While this study provides compelling theoretical and structural evidence regarding the conservation of the septin catalytic core, several inherent limitations must be explicitly noted:

Computational and In Silico Nature: This investigation is entirely computational and hypothesis-generating in nature. The docking metrics, structural superpositions, and molecular dynamics trajectories represent predictive models rather than direct biochemical binding measurements. Future in vitro and in vivo experimental validation—such as surface plasmon resonance (SPR), isothermal titration calorimetry (ITC), and cellular knockdown/overexpression invasion assays—is required to definitively confirm these functional interactions.
Pharmacological Specificity of the Ligand: Forchlorfenuron (FCF) was utilized in our molecular dynamics simulations strictly as a low-molecular-weight computational “molecular wedge” to evaluate target pocket stability. FCF is not a clinically validated, highly specific SEPT9 inhibitor and possesses well-documented, septin-independent off-target effects in living cells. Therefore, our computational drug-retention profiles should not be interpreted as definitive evidence of selective pharmacological efficacy or anti-metastatic therapeutic action.
Static Structures vs. Dynamic Cellular Environments: Our structural models rely on fixed crystallographic snapshots from the Protein Data Bank. Although molecular dynamics simulations were deployed to simulate structural flexibility over a dedicated timeline, these models cannot fully capture the real-time, macroscopic assembly, recruitment, and disassembly of higher-order septin hetero-oligomeric filaments within the complex, crowded cytoplasm of a living cancer cell.
Network and Database Confounders: The protein–protein interaction (PPI) networks derived from the STRING database encompass indirect associations, predicted links, and data generated via text mining. Furthermore, the clinical survival correlations extracted from the Kaplan–Meier Plotter database are strictly exploratory. These public datasets show highly probe- and cancer-type-dependent variations and do not account for all potential clinical, therapeutic, or molecular confounders, nor have they been corrected for multiple hypothesis testing.
Evolutionary Assumptions: The sequence space probability calculation (*p* = 20−15) demonstrates the extreme statistical improbability of random mutational convergence, strongly pointing to deep structural homology. However, this simplified mathematical model assumes a random mutational distribution and does not account for biological amino acid degeneracy, functional constraints, or complex structural selection pressures operating over evolutionary time.

## 4. Materials and Methods

The validity of utilizing an in silico framework to evaluate septin behavior is supported by the recent work of Grupp, Lemkul, and Gronemeyer [43], who successfully deployed computational modeling to determine inter-subunit affinities within human septin complexes. Our approach builds upon this precedent, utilizing thermodynamic docking to quantify the specific affinity of Forchlorfenuron (FCF; PubChem CID: 93379, National Library of Medicine, Bethesda, MD, USA) for the catalytic pocket. By centering our spatial grid on the conserved G1/P-loop region, we align our theoretical models with established computational benchmarks for septin–ligand interactions. To empirically validate the Fungal Island hypothesis and rigorously quantify the degree of evolutionary and structural conservation between metazoan and fungal septin engines, an extensive in silico bioinformatics pipeline was constructed. This multifaceted approach integrated primary sequence phylostratigraphy, heuristic multiple sequence alignment algorithms, 3D structural modeling, and thermodynamic molecular docking simulations. Initial sequence retrieval and pairwise alignments were executed programmatically utilizing the Biopython computational framework (Biopython Project, https://biopython.org) [44]. Sequence alignments and secondary structure topologies were rendered using the ESPript 3.0 web server (Institut de Biologie et Chimie des Protéines, Lyon, France) [45].

### 4.1. Primary Sequence Retrieval and Alignment Protocols

The FASTA-formatted sequences for the human septin target and its corresponding fungal ortholog were obtained directly from the UniProtKB database (UniProt Consortium, European Bioinformatics Institute, Hinxton, UK). The target for the human model was explicitly specified as SEPT9 isoform 1, identified by the unique UniProt accession number Q9UHD8. The fungal ortholog selected for comparative evolutionary analysis was the Saccharomyces cerevisiae cell division control protein 3 (Cdc3), identified by the accession number P32457. To accurately capture the evolutionary nuances between the human and fungal proteins, three distinct computational alignment paradigms were applied using the Bio.pairwise2 module:

Global Alignment (End-to-End Sequence Fitting): Executed via the pairwise2.align.globalxx algorithm, this method forced an end-to-end superposition to observe broad evolutionary divergence.

Local Alignment (Ancient Engine Localization): Executed via the pairwise2.align.localxx algorithm, this method searched exclusively for the highest-scoring sub-sequences without penalizing the highly divergent, non-homologous terminal regions. By ignoring the terminal noise, this algorithm successfully isolated the highly conserved inner catalytic core.

Threshold-Based Island Detection: Custom computational string-parsing logic was applied to systematically extract “Ancient Islands of Identity”. The mathematical threshold for a Fungal Island was defined as any continuous sequence of exact amino acid matches exceeding a length of five residues, uninterrupted by gaps or mismatches. While the search value for the threshold was 5 residues, the primary “catalytic” “island” used for the probability calculation reached a length of 15 residues.

### 4.2. Structural Modeling and Target Preparation

High-resolution three-dimensional structural models were acquired from the Protein Data Bank (PDB; Research Collaboratory for Structural Bioinformatics, Rutgers University, Piscataway, NJ, USA). To ensure high fidelity in our molecular dynamics and docking simulations, the human SEPT9 target domain was modeled utilizing the high-resolution apo crystal structure (PDB ID: 4YQF). To represent the highly conserved ancestral baseline and provide a comparative structural template within the human genome, human SEPT2 (PDB ID: 2QNR) was utilized. Prior to executing the molecular docking algorithms, both structures were meticulously processed to isolate the primary nucleotide-binding pockets by stripping extraneous heteroatoms, co-crystallized ligands (such as GDP), and solvent molecules to accurately calculate and assign Gasteiger partial charges to the target macromolecules.

### 4.3. Molecular Docking Simulations

Rigorous in silico molecular docking was performed utilizing AutoDock Vina version 1.2.0 (The Scripps Research Institute, La Jolla, CA, USA) [46], with an exhaustiveness of 32 to ensure comprehensive conformational sampling. To address the dynamic flexibility of septin filaments and empirically validate the stability of the drug-target complex, molecular dynamics (MD) simulations were conducted using the GROMACS package (GROMACS Development Team, University of Groningen, Groningen, The Netherlands). We analyzed the Apo and Holo states of both SEPT2 and SEPT9, measuring the radius of gyration (Rg), solvent accessible surface area (SASA), and root mean square deviation/fluctuation (RMSD/RMSF) of the P-loop and the ligand.

Furthermore, to explicitly differentiate deep homology from convergent evolution and address the broader systemic implications of the atavistic model, we executed a protein-protein interaction network analysis utilizing the STRING database API version 12.0 (STRING Consortium, Zurich, Switzerland). This pipeline mapped the interaction networks of SEPT9 and its ancestral templates to quantify the conservation of downstream migratory effectors.

### 4.4. Molecular Dynamics Simulation Protocol

To empirically validate the stability of the FCF-target complexes and assess the dynamic flexibility of the septin P-loop, high-throughput molecular dynamics (MD) simulations were executed using the GROMACS package (GROMACS Development Team, University of Groningen, Groningen, The Netherlands). The initial topology and coordinate files for both the Apo and Holo states of human SEPT2 (PDB ID: 2QNR) and SEPT9 (PDB ID: 4YQF) were meticulously constructed utilizing the CHARMM-GUI Solution Builder web-based interface (CHARMM-GUI Development Team, Lehigh University, Bethlehem, PA, USA) [47,48], employing the CHARMM36m all-atom force field [49] to accurately capture the dynamic behavior of the folded protein domains.

The simulation systems were prepared with the following specific parameters to mimic physiological conditions:

Protonation State: The protonation states of all titratable residues were determined and assigned at a physiological pH of 7.4. pKa calculations were verified to ensure accurate protonation prior to solvation.

Solvation Box: Each protein complex was centered within a rectangular simulation box with a minimum clearance distance of 10 Å between the protein surface and the box edges.

Ionization: The systems were neutralized, and the physiological ionic strength was calibrated using Monte Carlo ion placement.

Hydrogen Mass Repartitioning (HMR): To enhance computational efficiency and permit a larger integration time step, hydrogen mass repartitioning was applied.

Temperature Coupling: All production runs were maintained at a constant physiological temperature of 310.15 K.

Following energy minimization and rigorous NVT/NPT equilibration phases, production MD trajectories were generated. Post-simulation trajectory analysis was conducted using the native GROMACS analysis toolset [50]. Specifically, structural compactness and expansion were measured using *gmx gyrate* (radius of gyration) and *gmx sasa* (solvent accessible surface area). Backbone stability and residue-level flexibility were quantified using *gmx rms* (root mean square deviation) and *gmx rmsf* (root mean square fluctuation), respectively. Intermolecular hydrogen bonding networks between the FCF ligand and the P-loop were calculated using *gmx hbond*.

### 4.5. Sequence Space Probability Calculations

To mathematically distinguish between convergent evolution and deep homology, a sequence space probability model was utilized [51]. For a sequence of length *N* constructed from the 20 canonical amino acids, the total sequence space equals 20*^N^* possible configurations. The probability (*P*) of two distantly related lineages independently converging on the exact same amino acid sequence via random mutation is calculated as *P* = 20^(−*N*)^.

### 4.6. Three-Dimensional Structural Superposition and Image Analysis

All 3D structural superpositions and molecular visualizations were generated using the PyMOL Molecular Graphics System Version 2.5.4 (Schrödinger, LLC, New York, NY, USA).

### 4.7. Clinical Survival Analysis via Kaplan–Meier Plotter

To validate the clinical and prognostic relevance of SEPT9 overexpression in cancers characterized by high compressive and mechanical tissue stress, we analyzed patient survival data utilizing the Kaplan–Meier Plotter database (Semmelweis University, Budapest, Hungary) [52]. The analysis focused on four solid malignancies: colon, pancreatic, gastric, and lung cancer. For colon, gastric, and lung cohorts, microarray expression data spanning multiple SEPT9 probes (207425_s_at, 208657_s_at, and 41220_at) were evaluated. For the pancreatic cancer cohort, RNA-seq expression data were analyzed (n = 845). Patients were stratified into “high”- and “low”-expression cohorts using either the automatic best cutoff or median thresholds. The prognostic value was assessed using hazard ratios (HR) with 95% confidence intervals and log-rank *p*-values.

## 5. Conclusions

The findings presented in this comprehensive theoretical and computational analysis strongly substantiate the atavistic model of cancer progression. The exhaustive in silico molecular docking and sequence alignment data reveal that the human SEPT9 protein and its yeast ortholog Cdc3 share striking three-dimensional homology within their localized catalytic cores. By demonstrating a near-identical thermodynamic binding environment and profound structural conservation, the data provide compelling theoretical evidence that malignant tumors may undergo a reversion to deeply conserved “Fungal Islands” to facilitate physical invasion.

Because SEPT9 uniquely occupies the terminal positions in septin octamers [53] and mediates polymerization-dependent functions, disrupting its catalytic core has the potential to disable the entire ‘zipper’ mechanism of the atavistic engine. However, current pharmacological agents utilized to inhibit septin dynamics, such as forchlorfenuron (FCF), function as blunt instruments rather than precision tools; they are severely limited by off-target toxicities that perturb independent cellular signaling cascades rather than specifically engaging the septin apparatus [54].

Ultimately, recognizing these deep evolutionary homologies is critical for future pharmacological design. This highly conserved, immutable pocket serves as a rational structural template for next-generation therapeutic compounds. This evolutionary perspective supports moving oncology beyond the ‘search and destroy’ cytotoxic model toward a highly precise ‘paralyze and contain’ framework, halting systemic dissemination by locking the cancer’s mechanical engine in an immutable, non-functional state.

## Figures and Tables

**Table 1:** Molecular dynamics (MD) comparative summary of SEPT2 and SEPT9 (Apo and Holo states).

Metric	SEPT2 Unbound	SEPT2 Holo	SEPT9 Unbound	SEPT9 Holo
Mean Rg (nm)	1.797	1.823	1.799	1.788
P-loop RMSD (nm)	0.421	0.466	0.190	0.187
Ligand RMSD (nm)	n/a	3.426 (unstable)	n/a	1.109 (stable)
Ligand SASA (nm^2^)	n/a	7.11	n/a	7.08
H-bonds (ligand–P-loop)	n/a	0.0	n/a	1.76
Interaction energy (kJ/mol)	n/a	−110.3	n/a	−217.2
Protein SASA	127.6	131.5	140	135.8

**Table 2:** Summary of Kaplan–Meier (KM) data.

Cancer Type	Probe ID	Survival Metric	HR	*p*-Value	Finding
Colon	207425_s_at	RFS	N/A	0.007	Significant
Colon	208657_s_at	RFS	N/A	3.8 × 10^−6^	Highly significant
Colon	41220_at	RFS	N/A	0.0954	Not significant
Pancreatic	SEPTIN9	OS	1.28	0.0118	Significant
Gastric	207425_s_at	OS	N/A	7.7 × 10^−7^	Highly significant
Gastric	208657_s_at	OS	N/A	6.7 × 10^−9^	Highly significant
Gastric	41220_at	OS	N/A	1.7 × 10^−5^	Highly significant
Lung	208657_s_at	OS	N/A	0.0002	Highly significant
Lung	207425_s_at	OS	N/A	N/A	Significant
Lung	41220_at	OS	N/A	0.578	Not significant

Abbreviations: HR, hazard ratio; OS, overall survival; RFS, relapse-free survival; N/A, not available in the Kaplan–Meier Plotter output for the corresponding analysis.

**Table 3:** Phylostratigraphic evolutionary age estimates of the core cancer invasion apparatus.

Estimated Phylostratum (Evolutionary Age)	Cellular Invasion Function	Gene/Protein Hub
Pre-Metazoan/Opisthokonta (ps2-ps3)	Actin-bundling, GTPase engine, Nuclear protection	SEPT9
Early Eukaryota/Metazoa (ps1-ps5)	Orthogonal network scaffolding, Mechanosensing	FLNA
Early Metazoa/Eumetazoa (ps6)	Rho GTPase effector, Septin-Actin coordination	CDC42EP5

## Data Availability

The custom Biopython pipelines, gromacs trajectory files, sequence alignment algorithms, and spatial clustering code utilized to identify and analyze the Fungal Islands in this study are openly available at [https://github.com/ozcanomereren61-ui/In_vitro_Pipeline/tree/main (accessed on 12 March 2026)]. The docking simulations were performed using AutoDock Vina 1.2.5 with a search grid centered at x = 28.522, y = −14.398, and z = −32.512 for Human Septin9 (PDB ID 4YQF) and at x = 26.65, y = −14.77, and z = −32.56 for targeting the P-loop region of Septin2 (PDB ID 2QNR).

## Supplementary Materials

The following supporting information can be downloaded at: https://www.mdpi.com/article/10.3390/ijms27135743/s1.

## Abbreviations

The following abbreviations are used in this manuscript:

BORG3	Binder of Rho GTPases 3;
Cdc3	Cell division control protein 3;
ECM	Extracellular matrix;
FCF	Forchlorfenuron;
FLNA	Filamin A;
PDB	Protein Data Bank;
RMSD	Root-mean-square deviation;
SEPT9	Human septin 9;
SMT	Somatic mutation theory.
