# Supplementary material for: In Silico Identification of Conserved ‘Fungal Islands’ in Human Septin9: Evidence for Atavistic Therapeutic Targets
Source: Int J Mol Sci. 2026 Jun 25;27(13):5743. doi: 10.3390/ijms27135743 (PMC13362331; doi:10.3390/ijms27135743)
Supplement: Supplementary file 1 [file ijms-27-05743-s001.zip › ijms-4372835-supplementary.pdf]

Supplementary Table S1. Descriptive evolutionary annotation of selected SEPT9-associated cytoskeletal proteins.

Gene / protein	Main cytoskeletal or mechanobiological relevance	Descriptive evolutionary annotation
SEPT9	Septin GTPase; cytoskeletal organization; membrane compartmentalization; cell polarity; cancer-associated mechanical behavior	Evolutionarily conserved septin family protein with conserved GTPase-domain features
SEPT7	Core septin complex component; hetero-oligomer formation; structural organization of septin filaments	Evolutionarily conserved septin family protein involved in septin complex assembly
FLNA	Actin crosslinking; mechanotransduction; focal adhesion dynamics; migration-associated cytoskeletal remodeling	Metazoan cytoskeletal scaffolding protein associated with complex multicellular cell mechanics
CDC42EP5 / BORG3	Rho GTPase effector; actin-septin coordination; contractility and invasive cell behavior	Metazoan-associated cytoskeletal regulatory protein linked to polarity and migration pathways

Note: This table provides a descriptive evolutionary annotation based on available literature and database information. It should not be interpreted as an independent phylostratigraphic analysis unless a separate phylostratigraphy pipeline, gene-age assignment criteria, and validation strategy are explicitly provided.
